# Introme accurately predicts the impact of coding and noncoding variants on gene splicing, with clinical applications

**DOI:** 10.1186/s13059-023-02936-7

**Published:** 2023-05-17

**Authors:** Patricia J. Sullivan, Velimir Gayevskiy, Ryan L. Davis, Marie Wong, Chelsea Mayoh, Amali Mallawaarachchi, Yvonne Hort, Mark J. McCabe, Sarah Beecroft, Matilda R. Jackson, Peer Arts, Andrew Dubowsky, Nigel Laing, Marcel E. Dinger, Hamish S. Scott, Emily Oates, Mark Pinese, Mark J. Cowley

**Affiliations:** 1 Children’s Cancer Institute, Lowy Cancer Research Centre, UNSW Sydney, Sydney, NSW Australia; 2 School of Clinical Medicine, UNSW Medicine & Health, UNSW Sydney, Sydney, NSW Australia; 3 University of New South Wales Centre for Childhood Cancer Research, UNSW Sydney, Sydney, NSW Australia; 4 Kinghorn Centre for Clinical Genomics, Garvan Institute of Medical Research, Sydney, Australia; 5 Department of Neurogenetics, Kolling Institute, St. Leonards, NSW Australia; 6 Sydney Medical School-Northern, Faculty of Medicine and Health, University of Sydney, Sydney, NSW Australia; 7 Division of Genomics and Epigenetics, Garvan Institute of Medical Research, Sydney, Australia; 8 Clinical Genetics Unit, Institute of Precision Medicine and Bioinformatics, Sydney Local Health District, Sydney, Australia; 9 Centre for Medical Research, University of Western Australia, Harry Perkins Institute of Medical Research, QEII Medical Centre, Nedlands, WA Australia; 10 Department of Genetics and Molecular Pathology, Centre for Cancer Biology, An Alliance Between SA Pathology and the University of South Australia, Adelaide, Australia; 11 Australian Genomics, Parkville, VIC Australia; 12 Department of Genetics and Molecular Pathology, SA Pathology, Adelaide, Australia; 13 School of Biotechnology and Biomolecular Sciences, University of New South Wales, Sydney, Australia; 14 School of Medicine, University of Adelaide, Adelaide, SA Australia; 15 ACRF Cancer Genomics Facility, Centre for Cancer Biology, An Alliance Between SA Pathology and the University of South Australia, Adelaide, SA Australia

**Keywords:** Splicing, Variant interpretation, Deep intronic, Splice region, Splice site, Intronic variant, Splicing regulatory element, Genomics, Clinical genetics

## Abstract

Predicting the impact of coding and noncoding variants on splicing is challenging, particularly in non-canonical splice sites, leading to missed diagnoses in patients. Existing splice prediction tools are complementary but knowing which to use for each splicing context remains difficult. Here, we describe Introme, which uses machine learning to integrate predictions from several splice detection tools, additional splicing rules, and gene architecture features to comprehensively evaluate the likelihood of a variant impacting splicing. Through extensive benchmarking across 21,000 splice-altering variants, Introme outperformed all tools (auPRC: 0.98) for the detection of clinically significant splice variants. Introme is available at https://github.com/CCICB/introme.

**Supplementary Information:**

The online version contains supplementary material available at 10.1186/s13059-023-02936-7.

## Background

An important challenge in genomic medicine is the accurate identification of genetic variants that either cause disease or drive disease progression, and overcoming this challenge is critical to achieving a genetic diagnosis. The use of genome sequencing for rare genetic diseases can generally provide a diagnosis in 40–60% of cases [1]. These current diagnostic rates largely only consider coding, copy number, and canonical splice site variants. A key challenge in increasing diagnostic yield is in identifying splice-altering genetic changes and interpreting their functional impact. It is estimated that 9–30% of all disease-causing variants operate by impacting splicing [2–4], with one recent study finding that 75% of previously undiagnosed patients harboured pathogenic atypical splice altering variants [5]. Therefore, through the implementation and improvement of in silico splice-altering variant recognition, we expect to observe a substantial increase in diagnosis rates.

The process of splicing is critical for the accurate generation of mRNA and ultimately protein. The delineation of coding regions by the precise removal of intronic DNA from pre-mRNA is orchestrated by over 200 proteins and small nuclear RNAs (snRNAs) through the recognition of defined sequence motifs [6]. The main splicing motifs are the essential donor (5′) and acceptor (3′) splice sites at either end of the intron, the branchpoint, and the polypyrimidine tract (PPT) [6]. Additionally, there are regulatory elements, such as enhancers and silencers, in exons and introns that influence splice-site usage and exon inclusion [7]. Splice-altering variants can cause exon skipping, intronic read-through, cryptic exon inclusion, or shift the open reading frame to produce an aberrant gene product [8]. This can result in reduced or absent function at the protein level or complete loss of protein expression due to mechanisms such as nonsense-mediated mRNA decay [9]. However, splice-altering variants can be challenging to identify as a variant at any location in a gene has the capacity to affect splicing [6, 10]. Currently, many of these variants go unrecognised due to incomplete understanding of the complex splicing process and an absence of reliable analysis algorithms that can identify these variants.

Several in silico methods have been developed to predict the likely splicing-impact of a genetic variant [11–13]. Choosing which programs to run is challenging because most tools focus on specific regions or splicing motifs. Early splice prediction tools focussed on scoring potential RNA binding protein (RBP) sites, such as the 5′ and 3′ essential splice sites (MaxEntScan [14]), or exonic splicing enhancers (ESEs) (ESEFinder [15]). This category of predictive tool relies on prior knowledge of the often-degenerate binding motifs. More recently, composite splice predictors, such as dbscSNV [16], have combined multiple motif-specific methods to assess the impact of a variant on splicing. Since dbscSNV only focuses on previously known 5′ and 3′ splice sites, it has limited utility for evaluating de novo splice site creation and novel splice site usage. These motif-specific tools accurately identify splice-altering changes in their respective motifs; however, splicing is more complex than individual elements and a compartmentalized approach does not account for the interplay between motifs. Therefore, a more holistic approach that considers multiple factors, elements, and their spacing is required for more accurate splicing predictions.

Another category of in silico splice-prediction tools is overall predictors, which attempt to identify all types of splice-altering variants, often constructed using artificial intelligence methods. The first deep learning method, SPANR/SPIDEX [17], used RNA sequencing (RNA-seq) data to predict exon inclusion based on learned DNA sequence features. Subsequently, a deep learning tool called SpliceAI [3] was trained to recognise DNA elements important for splicing by learning this behaviour from the human reference genome sequence and comprehensive maps of known intron–exon boundaries. Other overall predictors include MMSplice [18], which used data from high-throughput perturbation assays to recognise the impact of variants on key splicing elements, and Spliceogen [19], which generates scores from multiple splicing tools, but only reports on a gain or loss of splice sites based on MaxEntScan scores. CADD-Splice [20] is a universal in silico functional effect predictor that simultaneously evaluates variants for their impact on protein coding sequence or splicing, but does not report whether high-scoring variants are due to their coding or splicing impact.

Here, we describe a novel in silico splicing analysis tool called Introme, which evaluates a variant’s likelihood of altering splicing by combining predictions from multiple splice-scoring tools, combined with additional splicing rules, and gene architecture features. Introme can accurately predict the impact of human coding and noncoding variants on splicing through investigating for the potential damage, creation, or strengthening of splice elements and outperforms all leading tools that we tested. This was achieved using a machine learning approach to optimise the performance of several best-in-class splice-detection tools. Introme allows the investigator to comprehensively identify splice-altering variants, avoiding the need to consult and interpret the outcomes from multiple tools.

## Results

### Assessment of existing splice-prediction tools

To guide the development of Introme, we undertook a comprehensive review of the literature to curate a list of variants functionally validated for splicing impacts using a variety of techniques, including RNA-seq, minigene splicing assays, and RT-PCR. We identified 1174 variants with and 611 variants without an effect on splicing across a range of rare genetic disease and cancer genes were used to train or test Introme (total *n* = 1785; Additional file 1: Table S1). The variants collected had varying modes of impact on splicing and over two thirds occurred outside of canonical splice sites.

**Fig. 1:**
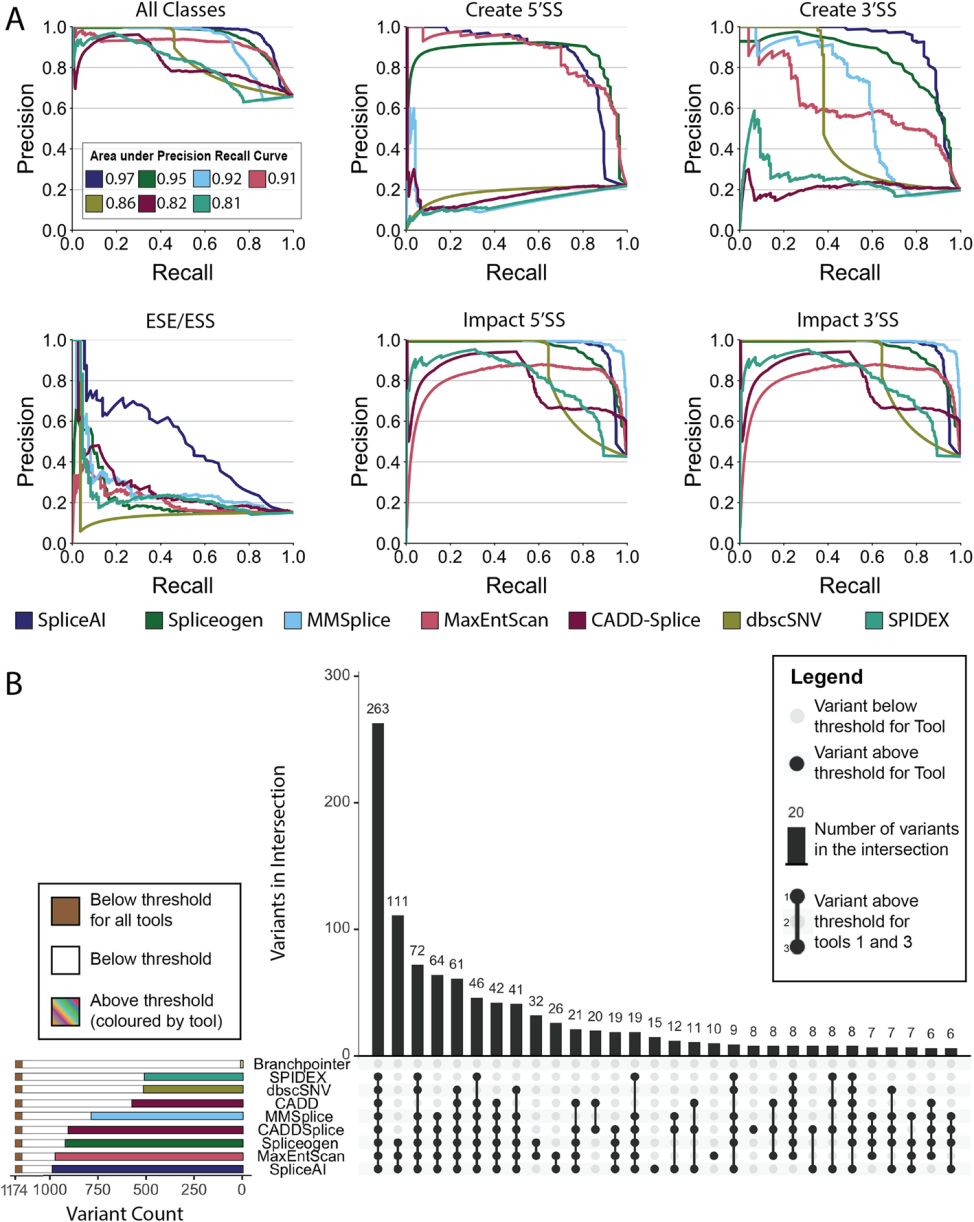
Assessment of existing in silico splice prediction tools on functionally validated splice-altering variants. **A** The performance of each tool is shown using precision recall curves (PRCs) of functionally validated splice-altering and non-splice-altering variants, grouped by the class of the variant. PRCs compare the precision (proportion of calls that are relevant) and recall (proportion of total relevant variants that are called) across different score thresholds, with better performing tools appearing near the top right. Variants that impacted the polypyrimidine tract or branchpoint were classified as impacting the acceptor splice site (3′SS). 5′SS: donor splice site; ESE/ESS: exonic Splicing Enhancer/Silencer. **B** Comparison of variants identified by splicing tools. The overlap of known splice-altering variants that are also predicted to be splice-altering by each in silico tool (i.e., above threshold) are represented as an UpSet plot [21]. For each comparison (the top 30 are shown), the number of predicted splice-altering variants (vertical bars) and the tool(s) that identified these variants (solid dots) are shown. The horizontal bars show the total splice-altering variant identification rate of each tool, coloured as per legend

We then tested multiple existing in silico splice prediction tools against our dataset, which revealed substantial differences in tool performance that was dependent on the class of splice variant (Fig. 1A). The performance of each tool was investigated across a range of splice-altering variant classes categorised by the effect the variant had on splicing. Variants that altered existing acceptor splice sites (3′SS) and donor splice sites (5′SS) were identified by most tools, with MMSplice and SpliceAI having the highest performance. However, variants which created new 3′SS or 5′SS were not identified consistently by all tools. As expected, tools which were precomputed around existing splice sites (dbscSNV, CADD-Splice, SPIDEX) were unable to identify variants which created novel splice motifs outside of their search space. There is a greater variability in tool performance on 3′SS creation variants. Notably, MMSplice had the strongest overall performance for variants impacting existing splice sites yet performed poorly on splice site creation. Variants altering exonic splicing enhancers (ESE) or exonic splicing silencers (ESS) were the most difficult for the programs to identify (Fig. 1A). Crucially, there was widespread evidence of tool specialisation, where each tool performed well on some types of splice changes and poorly on others. SpliceAI returned the highest overall area under the precision recall curve (auPRC); however, it ranged from 0.50 on ESE/ESS variants to 0.96 on 5′SS creation. Furthermore, MMSplice was the leading tool for identifying variants that impacted the 5′SS (auPRC: 0.99) or 3’SS (auPRC: 0.94), yet performed poorly on other categories, which affected its overall performance (auPRC: 0.92). Additionally, tools such as MMSplice, SPIDEX, and dbscSNV all have a limited location in a pre-mRNA in which they operate, leading to robust performance solely in those regions. Taken together, these results suggest that no single tool was superior for identifying splice-affecting variation in all contexts and that a general-purpose splice predictor would need to draw on the strengths of multiple tools.

As tool performance fluctuates depending on the type of splice-altering variant, it is common practice to combine multiple splice-prediction tools to seek consensus on a variant’s splice-altering potential. To assess the validity of this approach, we analysed the overlap of predictions on *n* = 1174 known splice-altering variants (Fig. 1B) and *n* = 611 variants without an effect on splicing (Additional file 2: Fig. S1). Most true splicing variants were detected by at least one tool (97%) at the recommended thresholds, but even the best performing tool found only 91% if used alone. Taking the variants found by the union of all tools resulted in a high false positive rate of 65%. Most of these false-positive predictions were made by a single program (42%). Taken together, these results suggest that although existing splice prediction tools are collectively able to identify a wide range of splice variant types, a sophisticated approach is required to combine the output of existing tools and achieve an optimised consensus call.

### Development of a new comprehensive splice-prediction tool—Introme

To develop a comprehensive splice-prediction tool, we first determined a comprehensive collection of features relevant for gene splicing. These included the scores from several leading splice prediction tools (as per Fig. 1B), ESE/ESS motif strength, as well as sequence features such as whether the variant is in the branchpoint region, the AG Exclusion Zone (between the branchpoint and the 3′SS) [22], in a U12 intron (~ 0.5% of all introns are excised by the minor spliceosome instead of the major spliceosome) [23], and if the variant would lead to an intron smaller than the minimal length intron (distance between the branchpoint and the previous 5′SS is < 45nt) [24] (see the “Methods” section and Additional file 2: Fig. S2). Following this feature selection, we selected a machine learning classification model that could handle missing data without imputation. We trained several models on the same training data, which were 80% of the variants that we curated from the literature (*n* = 940 with and *n* = 489 without functional impact), and when assessing model performance using tenfold cross validation, the C5.0 decision tree model [25] had the highest area under the receiver operating curve (auROC) and was thus selected as the basis for the Introme decision tree model (see the “Methods” section). The C5.0 learner has the added benefits of high-speed, and logical decision path (a series of nested if/else rules), which match the experience of investigators who may already be familiar with running each tool separately. The most important features in the model related to gene location, with scores from each of the different tools having varied contributions to the model (Additional file 2: Fig. S3), reinforcing our intuition from Fig. 1 that variant context is important for choosing the best splice-altering variant detection tool. Variants that affect splice branchpoint or U12 introns are rare, but biologically important, so despite these features having minimal overall importance, we retained these features in the model (Additional file 2: Fig. S3). This model returns a score from 0 to 1 per variant reflecting the likelihood that the variant affects splicing (hereafter the Introme score). Different use cases require varying levels of sensitivity and specificity; therefore, and based on the model performance in the validation data, we recommend two thresholds for Introme: the optimal balance between precision and recall (F1 Score) for Introme is at a threshold of 0.61, producing a sensitivity of 0.91 and a specificity of 0.91. When higher specificity is required, a threshold of 0.83 results in a sensitivity of 0.8 and a specificity of 0.975.

**Fig. 2:**
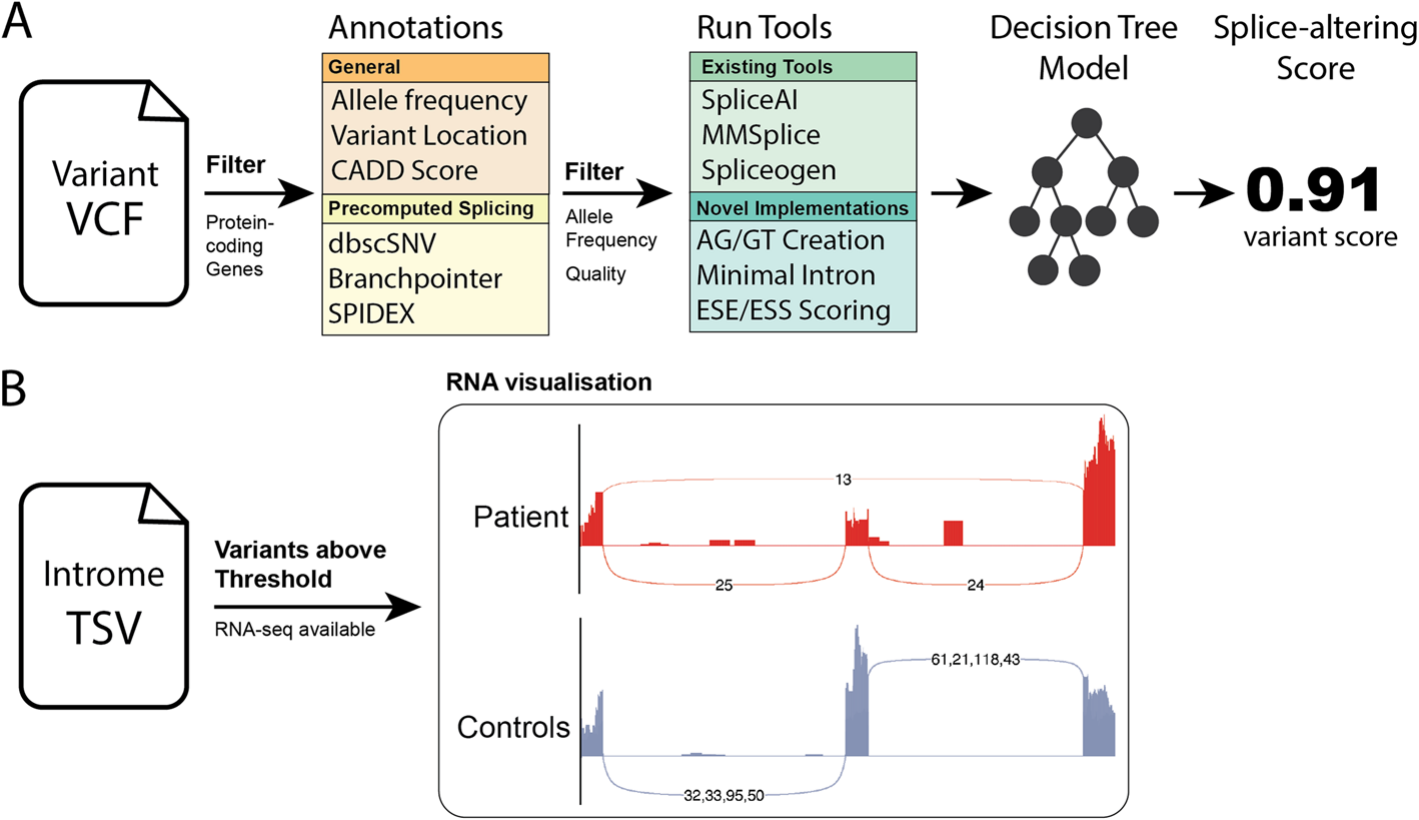
Introme’s pipeline for identifying splice-altering variants. **A** Introme’s scoring process from an input VCF, variants from protein coding genes are filtered before annotating. Variants are subsequently filtered based on populational allele frequency, scored through several splice prediction tools, before feeding through the Introme decision tree model to give a final splice-altering score. **B** If a variant has a score above the Introme threshold, a sashimi plot of the region can be generated using ggsashimi [26] if a complementary RNA-seq BAM file is available

To facilitate the use of this splice variant prediction system, we next developed Introme as a standalone software utility for evaluating the impact of any coding or noncoding variant(s) on splicing and enabling clinical interpretation. Introme takes a VCF file as input, which is then filtered to contain only variants within known protein-coding genes and below a defined population minor allele frequency (default 1%). The remaining variants are then automatically annotated with general variant information and the features (Fig. 2A). These features are then fed into the Introme decision tree model which returns the Introme score. Additionally, to facilitate the validation of splice-altering events, an RNA-seq BAM file can be provided, and any prediction above a user-specified threshold (default 0.61) will result in an automatically generated sashimi plot of the affected region. This produces a short-list of candidate splice-altering variants to be visualised and enables rapid confirmation of splice-altering variants. Additional gains in utility are seen due to Introme’s unique ability to evaluate the splicing impact of multinucleotide variants (MNVs) and simultaneous insertions and deletions (insdels) (see the “Methods” section).

### Assessing Introme’s performance against existing tools

**Fig. 3:**
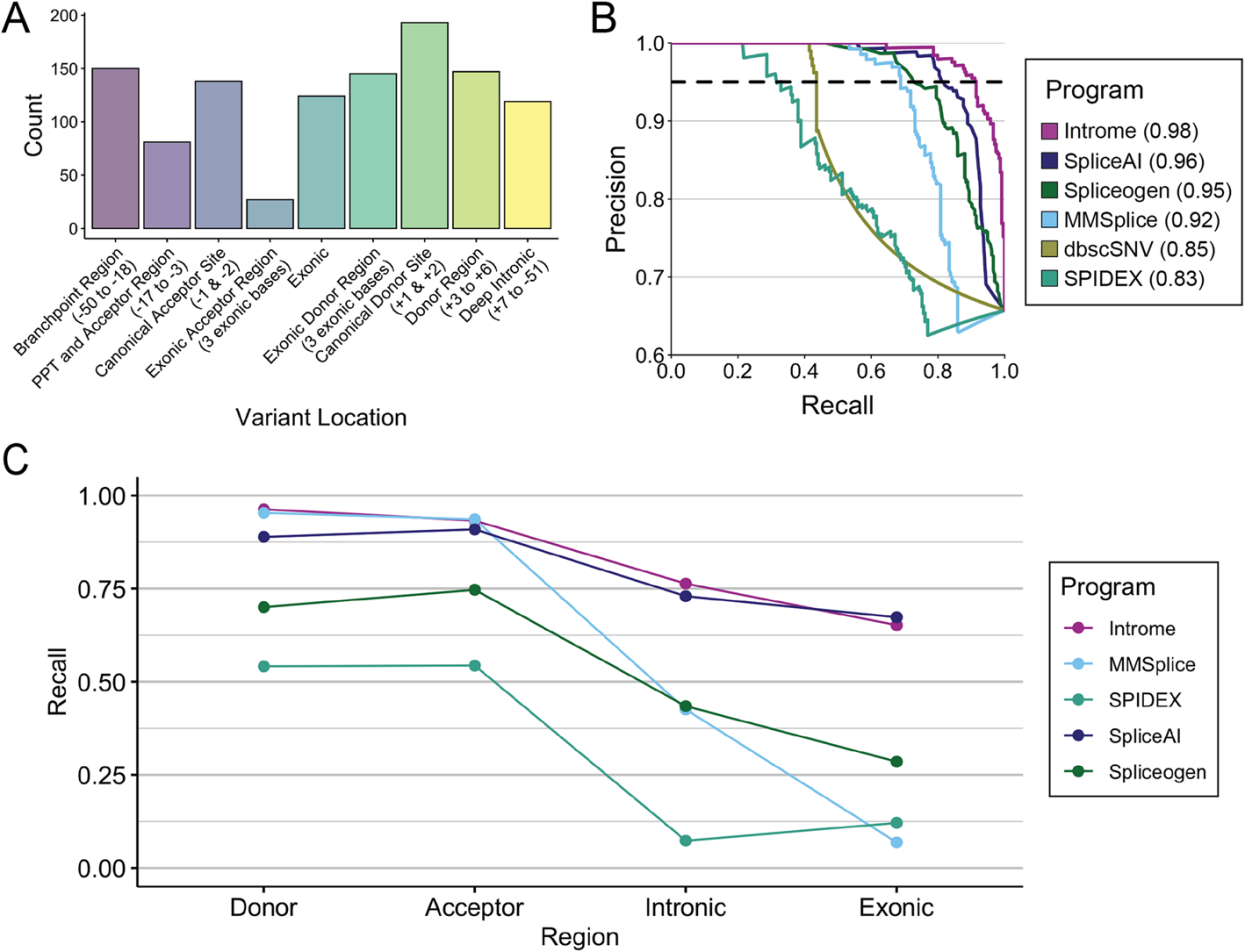
Introme performance comparison. **A** Breakdown of the location of splice-altering variants making up the validation set. The distance of the regions to respective intron–exon boundaries is in brackets. **B** Precision recall curve of several splice predictor programs on the validation set. The area under the curve (auPRC) for each tool is shown in the legend. The dotted line at 0.95 precision indicates the threshold used to evaluate performance in **C**. **C** Performance recall for each evaluated program across the multiple datasets by variant region. Thresholds used represent 0.95 precision on the validation dataset

To assess Introme’s performance in relation to several existing splice predictors, each tool was assessed on multiple datasets of splice-altering variants (Fig. 3). The primary dataset used for this comparison was the remaining 20% of variants (*n* = 356; *n* = 234 with, and *n* = 122 without functional impact) from the dataset that we curated from the literature and which were not included in the training set (see the “Methods” section). This validation set had variants distributed across all classes of splice-altering variants and regions with > 70% of variants outside the essential splice sites and was therefore considered to be a good dataset to measure overall program performance (Fig. 3A). On the validation set, Introme achieved the greatest performance (auPRC: 0.98), followed by SpliceAI (auPRC: 0.96) and Spliceogen (auPRC: 0.95) (Fig. 3B).

To further evaluate Introme, we performed additional benchmarking against three additional datasets of splice-altering variants (see the “Methods” section), collectively with 19,842 variants (Additional file 2: Fig. S4). Each dataset had strikingly different composition of splice-variant types (Additional file 2: Fig. S5A,C,E), helping to support a robust evaluation of the performance of splice altering tools. When evaluating the splice altering potential of a novel variant, the investigator does not know the class of the splicing impact (as shown in Fig. 1A), so we investigated performance with respect to the region that the variant falls in: donor, acceptor, exon, and intron. For each tool, we identified the threshold that gave a precision of 0.95 on the validation dataset (see the “Methods” section). Tools that did not achieve a 0.95 precision (i.e., dbscSNV) on the validation dataset were excluded from further analysis. Introme achieved the greatest recall on variants in all regions apart from exonic, both overall (Fig. 3C) and when considering each validation dataset independently (Additional file 2: Fig. S5B,D,F). All tools performed at their best when predicting the impact of variants the donor and acceptor splice regions; MMSplice again slightly outperformed SpliceAI and Spliceogen in the splice donor or acceptor regions but performed poorly on intronic and exonic variants (Fig. 3C and Fig. S6).

**Fig. 4:**
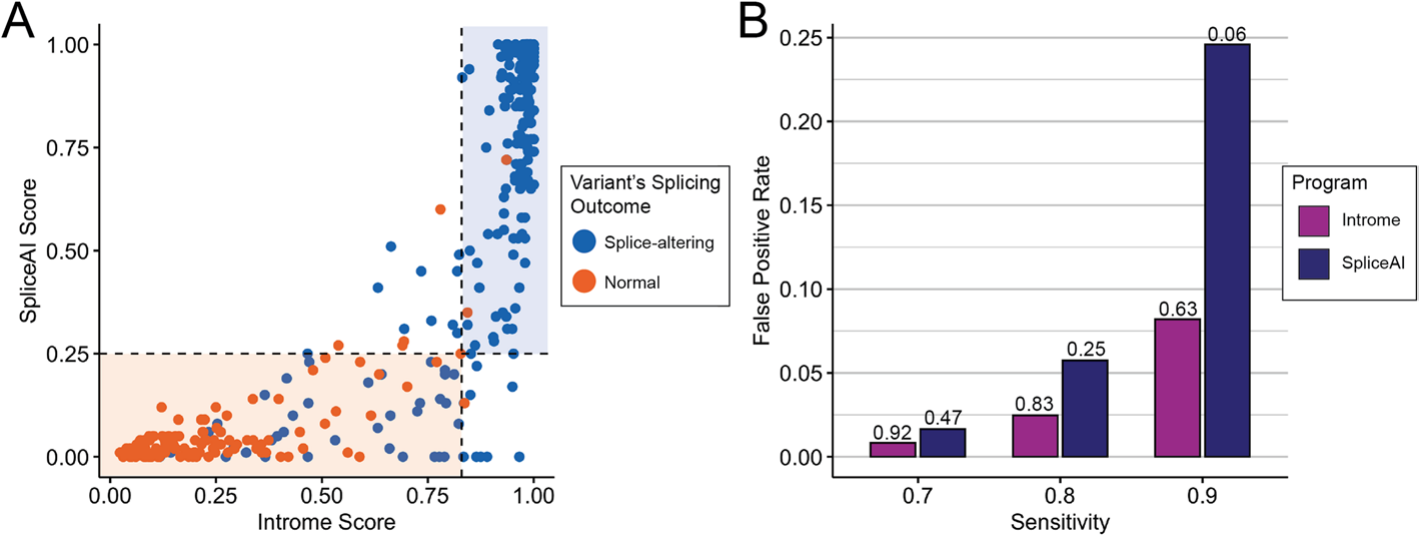
Comparison of the two top-scoring splice prediction tools: Introme and SpliceAI. **A** Introme and SpliceAI score comparison for validated splice-altering variants (positive held-out validation set—blue) and validated non-splice-altering variants (negative held-out validation set—orange). Thresholds represented by dotted lines (Introme: 0.83, SpliceAI: 0.27) correspond to a sensitivity of 0.80. Shaded regions represent the regions with both predictors classifying a variant as splice-altering (blue) or both classifying a variant as not affecting splicing (orange). **B** The false positive rates for Introme and SpliceAI at a range of sensitivities. Corresponding thresholds at a given sensitivity are shown above the bar

We compared the performance of Introme and SpliceAI, the second-best scoring tool, to investigate their performance in more detail. Using the held-out validation set, the majority (93%) of predictions made by Introme and SpliceAI were concordant, but of the discordant variants, Introme predicted fewer false positives than SpliceAI (3 vs 6) (Fig. 4A). This trend of reduced false positive predictions made by Introme was consistent at multiple sensitivity levels (Fig. 4B). A low false positive rate is beneficial in clinical settings, where high-confidence predictions are necessary to ensure the correct variants are being prioritised.

### Assessing Introme’s utility for variant discovery

**Fig. 5:**
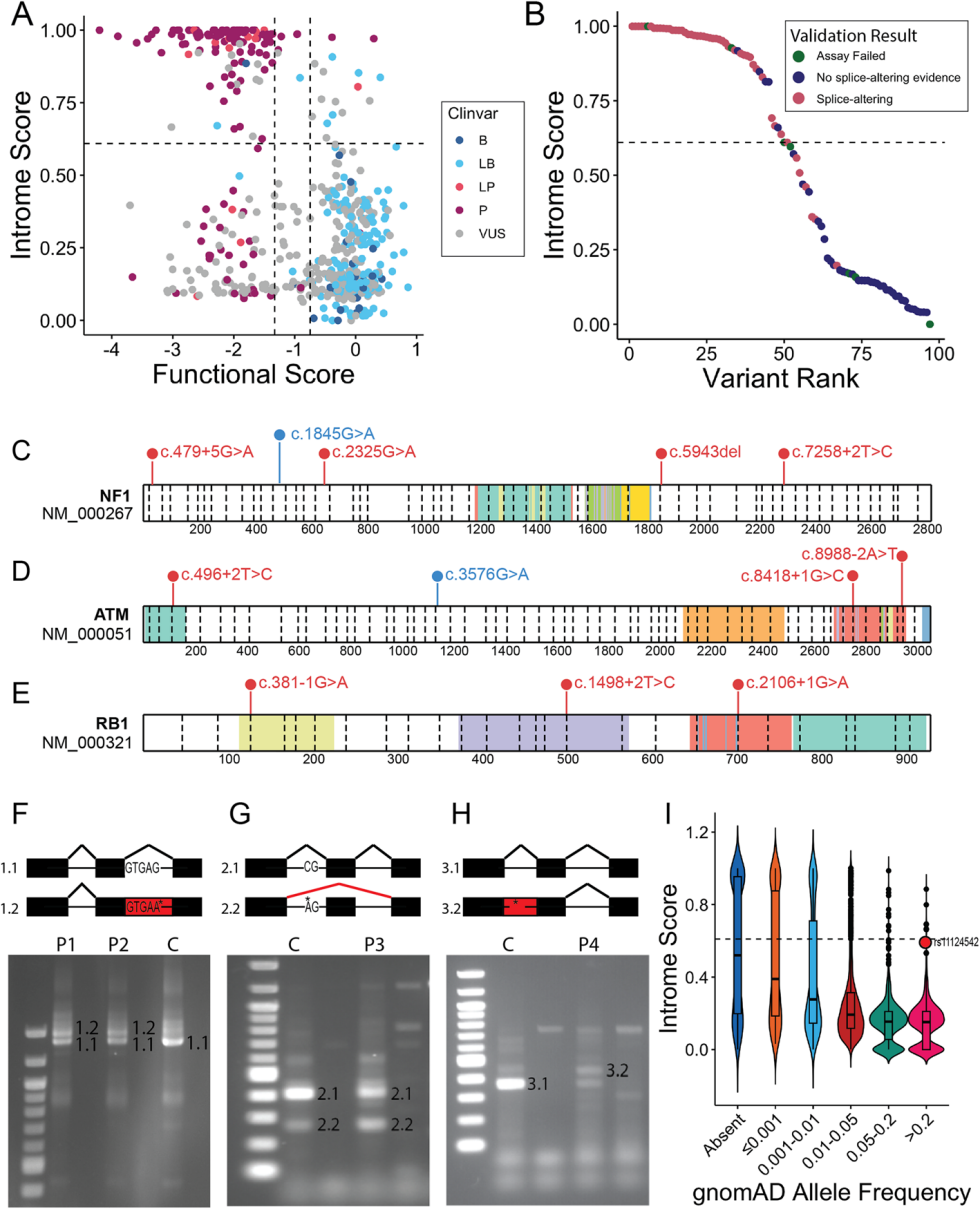
Introme performance in clinical applications. **A** The Introme scores and the functional scores calculated for *BRCA1* variants assessed using a saturation mutagenesis screen [27]. Variants with functional scores >  − 0.748 are classified as tolerated whilst functional scores <  =  − 1.328 correspond to non-functional variants, as determined in the original study [27]. Variants are coloured by their ClinVar pathogenicity classifications (P: pathogenic; LP: likely pathogenic; VUS: variant of uncertain significance; B: benign; LB: likely benign). **B** Introme scores of variants tested for splice-altering impacts by RT-PCR in a diagnostic lab. Variants were classed as ‘assay failed’ if the RT-PCR validation was not able to be performed on the sample. **C**–**E** Lollipop plot of the splice-altering variants identified by Introme in the ZERO childhood cancer cohort [28] for genes **C** *NF1*, **D** *ATM*, and **E** *RB1*, made using ProteinPaint [29]. Each circle represents the location of at least one genetic variant identified as reportable and splice-altering in the ZERO cohort (red: somatic variant, blue: germline variant). Shaded areas on the transcript represent protein domains. **F**–**H** Splice-altering variants in *PKD1* identified using Introme in patients with polycystic kidney disease. RT-PCR was performed using patient (P) and control (C) samples, with the corresponding transcripts numbered and represented above the gels. Asterisks mark the location of the variants in the diagram. The variants are **F** *PKD1*:c.7489 + 5G > A, **G** *PKD1*:c.11014-10C > A, and **H** *PKD1*:c.10167 + 25_10167 + 43del19. **I** A violin plot showing Introme scores for 7200 randomly selected variants with different population allele frequencies. In **A**, **B**, and **I**, the horizontal dashed line is the default Introme score threshold of 0.61

The recent development of high-throughput saturation mutagenesis screens which exhaustively mutate and assess the functional impact of all possible genetic variants within a defined genomic search-space represent a compelling opportunity to further benchmark Introme’s performance. We used a previously published saturation mutagenesis dataset [27] which comprehensively screened variants in all coding and 12 bp of intronic bases in 13 exons of *BRCA1*. The variants categorised both as non-functional and with a 75% reduction in mRNA were used as a truth set of splice-altering variants (*n* = 130) (see the “Methods” section). Introme was able to demonstrate that it can be used to identify the splice-altering variants from this dataset with minimal false positive predictions (auPRC of 0.96). When comparing the variants’ Introme score and their functional score, the variants cluster well according to their assigned ClinVar pathogenicity (Fig. 5A). Additionally, the two pathogenic variants with normal functional scores predicted as splice-altering by Introme result in in-frame splicing changes, as identified in the original study.

To demonstrate the performance of Introme on variants identified in a clinical context, we investigated 97 consecutive variants that had been referred to a diagnostic laboratory for clinically accredited RT-PCR testing (Additional file 1: Table S2). These variants had a suspicion of pathogenicity and could thus demonstrate the expected performance of Introme in a clinical laboratory situation. Introme correctly identified 78% of the RT-PCR validated splice-altering variants using the stringent threshold of 0.83, and 88% of these at the more relaxed threshold of 0.61 (Fig. 5B). There were three variants with an Introme score ≥ 0.83, but without supporting RT-PCR evidence (blue dots in Fig. 5B). For one variant, we prompted a re-review of the RT-PCR evidence, which revealed a clinical false negative that was missed during the initial interpretation of the RT-PCR data; this resulted in a reclassification of the variant to likely pathogenic. Additionally, the RT-PCR assay failed for seven of the 97 variants tested due to sample or laboratory issues (green dots in Fig. 5B), three of which Introme predicted would alter splicing and would therefore be worth additional effort to validate splicing abnormalities.

During the development of Introme, we used it to discover splice-altering variants in several studies. We identified 28 reportable (pathogenic or likely pathogenic) mostly somatic splice-altering variants in a cohort of 247 patients with high-risk paediatric cancers [28], notably in the well-established cancer genes *TP53*, *NF1*, and *RB1* (Fig. 5C–E). Additionally, we identified a 3′ UTR variant in *KLHL40* in a patient with nemaline myopathy 8 [30] and a missense variant in *YARS2* in a patient with a mitochondrial disorder known as MLASA2 [31], both of which Introme helped prioritise as candidate splice-altering variants. Here, we investigated three variants in four probands with suspected autosomal dominant polycystic kidney disease, who remained undiagnosed following clinical whole genome sequencing [32]. In two siblings, Introme classified *PKD1*:c.7489 + 5G > A [32] as splice altering (Introme score 0.98) resulting in intron retention, which was validated using cDNA Sanger sequencing (Fig. 5F; see the “Methods” section). Furthermore, Introme correctly classified an intronic variant *PKD1*:c.11014-10C > A as creating a new AG motif in the AG exclusion zone (Introme score 0.92) that caused exon skipping (Fig. 5G), and an intronic deletion, *PKD1*:c.10167 + 25_10167 + 43del19 (Introme score 0.78), that resulted in intron retention (Fig. 5H).

Finally, whilst the primary goal of Introme is to identify rare, splice-altering variants, we evaluated whether it could identify polymorphisms that might also affect splicing and potentially predisposition to common diseases. Thus, we ran Introme on a random selection of commonly genotyped polymorphisms chosen from six different population allele frequency tranches. As the polymorphisms became more common, they were less likely to be predicted by Introme as candidate splice-altering variants (Fig. 5I). Of the 1200 randomly selected variants with a population allele frequency > 20%, eight were predicted to be splice-altering (Introme ≥ 0.61). We manually inspected the top-scoring common variants for GWAS hits and found that rs11124542 was associated with Vitrin level in chronic kidney disease with hypertension and no diabetes (*p*-value 5 × 10^−12^, beta value 0.43 [0.31–0.55 CI]) [33]. Whilst most polymorphisms are not expected to be causal variants, in this instance, the variant (*VIT*:c.679 + 11A > C) is in the gene that encodes *Vitrin* and may be the causal variant through altering gene splicing, though further validation is required to validate this.

Taken together, these results demonstrate that Introme outperforms all tools tested when considering 21,000 true positives from four different validation datasets, one saturation mutagenesis screen, clinical data, and common polymorphisms.

## Discussion

We present Introme, a comprehensive method to predict splice-altering variants with several clinical applications. Introme draws from multiple class-leading splicing prediction tools, adds additional functionality to identify variants missed by existing tools, and simplifies the process of running and interpreting the results from each of the incorporated predictive tools. Introme evaluates variants at any location within a gene for splice-altering potential, from exonic to deep intronic variants. When compared with established splice-prediction tools, Introme achieved the best performance on a dataset of functionally validated variants with an auPRC of 0.98 and consistently outperformed the next best tool’s (SpliceAI) sensitivity and false positive rate across multiple validation datasets and gene regions. In addition, Introme’s ability to accurately classify variants and predict likely splicing outcomes was applied to several clinical datasets and showed utility for the identification of splice-disrupting genetic variants in several clinical settings. Introme has been used to discover splice-altering variants in both germline and somatic tissues, in childhood cancer [28], and several rare genetic diseases, including polycystic kidney disease (this study), neuromuscular disorders [30], mitochondrial disorders [31], dilated cardiomyopathy, epilepsy, and Parkinson’s disease (data not shown).

We have demonstrated that existing splice prediction tools are complementary, with distinct strengths and weaknesses. Prior to the development of Introme, investigators needed to learn these characteristics, and determine which tools to trust for each class of splicing variant, which made it challenging and time consuming to screen variants for splice-altering potential. Introme significantly lowers the barrier to entry to predicting the splicing impact of genetic variants, because it can detect all types of splice-variants in coding and noncoding sequences and has learnt where its constituent tools perform well. Introme’s use of a consensus scoring approach allows shortcomings of the constituent tools to be learnt and supplemented by other tools. For example, Introme relies heavily on SpliceAI, but for variants that impact existing canonical splice sites, the better performance of MMSplice and Spliceogen in these regions improves the overall performance of Introme. Furthermore, as SpliceAI was trained on naturally occurring intron–exon junctions, several edge cases, such as AG-exclusion zone variants, minimal-length introns, and splice junctions from the minor spliceosome can be mis-classified. We addressed these limitations not captured by any tool by adding additional rules. When SpliceAI and MMSplice do not score a variant, which we saw primarily in the common variant analysis (Fig. 5I), we observed a higher false positive rate. This was due to hard-coded region filters in these tools, so to address this, we changed the default behaviour of Introme to produce a score of zero in these cases. Because of the modular way that Introme has been developed, we anticipate adding more modules and rules over time to improve its performance.

Further research to predict the functional impact (e.g., intron retention, exon skipping) of splice-altering variants and improved methods to identify splicing regulatory variants are required to further improve the utility of in silico splice predictors. In our training data, only 10% of variants had a reported quantitative value for the impact on splicing, namely the percent spliced in (PSI) value. The field needs to first gather more data linking genetic variants, to functional splice alterations and PSI, perhaps via massively parallel splicing assays [34]; this would then allow tools to be developed that better predict the precise functional impact of a splice-altering variant on the resulting mRNA transcript. In support of this goal, initiatives like SpliceVault are shedding light on rates of naturally occurring atypical splice site usage [35]. Furthermore, the main category of splice-altering variants that Introme underperforms on are splicing regulatory elements such as exonic/intronic splicing enhancers and suppressors, as none of the tools that we evaluated predicted these variants with high accuracy. Two examples highlight the difficulty of interpreting splicing regulatory variants: first, a deep intronic SNV both damaged an ESS and strengthened an ESE, resulting in the expression of a deep intronic pseudoexon [36], and another variant which strengthened a cryptic splice site and several ESE elements, resulting in the splicing of two different pseudoexon isoforms [37]. Developing better tools to identify these variants may need to incorporate the strength and spatial organisation of several splicing regulatory elements. Despite these difficulties, Introme annotates variants with the gain/loss of several ESE/ESS elements (see the “Methods” section), which can identify variants warranting further investigation.

To develop Introme and benchmark it with other in silico prediction tools, we surveyed the literature to identify a large resource of variants with known splicing impacts. We aimed to identify variants across a whole range of classes, with a particular focus on identifying deep intronic and non-canonical splicing variants. We further supplemented this curated resource with three larger-scale datasets, primarily derived from matched exome and RNA-seq data, which thus have an ascertainment bias towards coding variants and regions directly surrounding the exon–intron boundary. This benchmarking again highlighted significant differences in the performance of splicing detection tools, with Introme consistently outperforming all other tools, and halving the false positive rate of SpliceAI. To our knowledge, this, and the subsequent analysis of clinical variants, represents the most comprehensive benchmarking of splicing tools and likely reflects the diversity of variants that will be identified in real-world setting. Despite these efforts, there are relatively few examples of some splice altering variants (like those that affect the minimal intron length, branchpoints or a U12 splice site), so more examples will need to be curated to fully evaluate performance in these difficult regions.

High-throughput validation methods such as saturation mutagenesis screens are powerful, but currently only a few genes have been investigated in such depth. Additionally, as demonstrated with the *BRCA1* screen, certain regions are prioritised to be screened, and often this excludes the vast majority of intronic material. Nevertheless, the results for *BRCA1* ClinVar variants (auPRC: 0.96) suggest that accurate in silico analysis of splice-altering variants in any gene is feasible using such a method.

The assessment of Introme on 93 consecutive variants submitted by a diagnostic laboratory reflects a common scenario where clinical genome analysts have carefully selected variants of uncertain significance that require additional functional validation prior to reporting. Designing RT-PCR assays to validate the impact of a variant on splicing demands accurate prediction as to the likely functional impact of the variant on intron retention or exon skipping to know where to design primers. In situations where a high Introme score is obtained but is not supported by evidence from RT-PCR, careful review of assay design and results may be warranted, particularly where only one allele may be under investigation. We also showed that the splicing investigation of rare variants in *PKD1* could identify atypical splice-altering variants that were overlooked by clinical whole genome sequencing, and subsequently validated using cDNA Sanger sequencing.

## Conclusions

Due to the large number of sequence elements and splicing factors involved, predicting the impact of a genetic variant upon splicing can be difficult. Introme combines some of the best splice-prediction tools available using a decision tree model that achieved an auPRC of 0.98. Introme proved to be of most value when high-confidence predictions were required, achieving a sensitivity of 0.89 at 0.98 specificity. Improved methods for the detection of splice-altering variants combined with the increased use of genome sequencing or targeted sequencing with inclusion of intronic sequences will lead to more splice-altering variants being identified and implicated in disease.

## Methods

### Dataset preparation

Published variants that had been functionally tested for splicing abnormalities were collected and sorted into (1) those with a demonstrated impact on splicing (true positives) and (2) those with no impact on splicing (true negatives). Functional confirmation included minigene assay, patient-derived RNA-seq, or cDNA analysis. For a variant to be considered as splice-altering, it must be demonstrated to either produce a novel transcript or alter the ratio of existing transcripts compared to the sequence without the variant. If quantification of the transcripts was performed, the transcript(s) with altered splicing required a 10% change to be considered as splice-altering.

### Machine learning

Machine learning methods were applied using the “caret” R package (R version 4.0.4) [38]. Models that could handle missing data predictions without imputation were selected and each model was trained on a dataset of 1429 variants (training set), representing 80% of the 1785 functionally validated splice-altering variants collected from the literature. Tenfold cross validation was used to determine which tuning parameters resulted in the best performance for each model. The models assessed were CART: package “rpart” [39], C5.0: package “C50” [25], and AdaBoost and AdaBag: package “adabag” [40]. The C5.0 decision tree model produced the best auROC on the training data as determined by the tenfold cross validation and was thus selected as the model for Introme. Feature importance was calculated using the varImp caret function.

### Introme scoring

Introme reports on a variant’s likelihood of altering splicing by combining predictions from splice-scoring tools and general variant information with machine learning methods (Fig. 2). The input for Introme is a VCF file with GRCh37 or GRCh38 as the reference genome. Introme first subsets the file to contain only variants in the regions of interest (provided BED file or GTF). Remaining variants are then optionally filtered using user-provided quality metrics, annotated for population allele-frequency (both gnomAD [41] and MGRB [42]) using vcfanno [43], and, by default, filtered on gnomAD_PopMax_AF ≤ 0.01. Variants are then annotated using CADD V1.3 (Combined Annotation Dependent Database) [44], SPIDEX V1.0 (Splicing Index) [6], dbscSNV V1.1 (Database of single nucleotide variants within splicing consensus regions) [45], and Branchpointer (gencode_v26) [46]. The filtered variants are scored using SpliceAI V1.3 with a distance setting of 1000 (-D 1000) and no masking (-M 0). MMSplice_MTSplice V2.2 and Spliceogen (v2.0) scores are obtained using default parameters. Introme’s novel functions (detailed below) are then applied. The scores obtained by each individual tool are reformatted to a VCF-like format that is compatible with vcfanno. All generated scores are then annotated onto the initial VCF, converted to a TSV file, and scored using the Introme model in R. All Introme features and inclusions to the final Introme score are listed in Additional file 1: Table S3.

### Novel Introme functions

To further improve the ability of Introme to identify splice-altering variants, we developed several novel features focussed on known splicing rules (Additional file 2: Fig. S2). To score variants affecting the five main exonic splicing enhancers, SRSF1, SRSF1 (igM-BRCA2), SRSF2, SRSF5, and SRSF6, and the main exonic splicing silencer, hnRNP A1, we first obtained PWMs from ESEFinder [15] and experimental individual-nucleotide resolution crosslinking immunoprecipitation (iCLIP) data [47], respectively. Introme includes a PWM scoring script to assess the impact of a variant on each of these motifs.

To support the identification of splice-altering variants outside of the canonical splice regions, additional sequence features for the AG Exclusion Zone, U12 Spliceosome, and Minimal Introns were implemented. Variants which create or remove an AG or GT sequence were flagged—this is particularly of use in the AG exclusion zone. Annotations from the Intron Annotation and Orthology Database [48] were added to support the identification of variants present in introns where splicing is mediated by the minor spliceosome (U12), which are often overlooked due to their rarity. When an intronic deletion occurs, the intron length is calculated to capture variants which result in introns under the minimal length (45 nucleotides) [24].

To enable SpliceAI scoring for indels and multinucleotide variants, each complex variant was split into the corresponding deletion and insertion. The maximum score for each SpliceAI category was taken and used as the score for the complex variant.

### Comparison methods

The MFASS dataset [34] (*n* = 1050) was generated from a massively parallel splicing minigene reporter assay that focussed primarily on exonic variants and was sensitive to small changes in exon usage; therefore, the MFASS dataset is enriched for variants that affect exonic splicing enhancers or silencers (ESE/ESS). The ncVarDB dataset [49] (*n* = 536) contains variants identified from publications and may include noncoding variants that do not affect splicing. Not all variants in this dataset have been functionally validated, and we used the author assertions regarding each variant’s pathogenicity. The Shiraishi dataset [50] (*n* = 13,999) was derived from paired tumour exome and RNAseq data from The Cancer Genome Atlas (TCGA) [51] derived from dozens of cancer types and as such is enriched in coding and canonical splice sites; thus, the majority of tools should perform well on this dataset. The Shiraishi dataset was used to train Spliceogen, which may inflate performance for this tool on this dataset, as well as potentially Introme, which incorporates Spliceogen as one of its predictors.

The splice-altering variants from MFASS (dPSI >  = 0.5) [34], ncVarDB [49], and Shiraishi [50] datasets were grouped based on the location of the variant. Categories used to define variant groupings were donor (3 nt at 3′ of the exon and the contiguous 6 nt into the intron, forming the donor site), acceptor (3 nt at the 5′ of the exon and the preceding 12 nt into the intron forming the acceptor site), and classification based on intronic or exonic if not a donor or acceptor variant. True negative variants used for the comparison are sourced from MFASS (dPSI score < 0.1) and were sorted into the same categories. The performance of each tool was then assessed per category using a threshold corresponding to a precision of 0.95 on the validation data set. A new threshold was defined for each program to ensure a fair comparison based on the same precision score and dataset.

### Validation methods

Variants used for the saturation mutagenesis comparison were sourced from a study that assayed 96.5% of all possible SNVs for 13 exons in *BRCA1* [27]. Each variant was assigned a functional score (level of deleteriousness) and an RNA score (level of mRNA) in the original study. The variants categorised as non-functional (functional score ≤  − 1.328) with 75% reduced levels of mRNA (RNA score ≤  − 2) were used as a truth set of splice-altering variants. Nonsense variants in the dataset were removed from the analysis. The ClinVar categories were reported in the original study.

Four patients with suspected autosomal dominant polycystic kidney disease underwent clinical whole genome sequencing to at least 30 × depth, at Genome.One (Sydney, Australia), and raw data was released for research use. Patients were consented for research use and analysed as described previously [32].

Variants used for allele frequency assessment were obtained from Illumina’s InfiniumCore-24v1.2 BeadChip and sorted into six tranches based on gnomAD PopMax allele frequency [41]. A random set of 1200 variants for each tranche were selected, then scored using Introme (Additional file 1: Table S4).

## Supplementary Information

**Additional file 1: Table S1.** Experimentally validated variants from SpliceVarDB used to train and test Introme. **Table S2.** Variants experimentally validated using RT-PCR for splice-altering changes by SA Pathology. **Table S3.** Features in Introme. **Table S4.** Variants used for allele frequency assessment.

**Additional file 2: Figure S1.** Comparison of in silico splice-prediction tools on validated non-splice-altering variants. Using an UpSet plot, variants identified here by one or more tools are in silico false positives. The CADD and CADD-splice tools contributed the largest number of false positives. **Figure S2.** Novel Functions incorporated into Introme. A: Position Weight Matricesfor the exonic splicing enhancer motifs from ESEfinder [15] showing relative representationof nucleotides at each position of the motif. B: PWM for the hnRNP A1 exonic splicing silencer motif [50]. C: Normal splicing pattern with the blocks representing the location of the exons. Creation of an AG motif greater or equal to 15 nucleotides away from the branchpoint results in the use of the new splice site. Creation of an AG less than 15 nucleotides away from the branchpoint results in exon skipping due to the AG exclusion zone. The consensus motifs for exon definition of the minor spliceosomeare different than the major spliceosome. Intronic deletions which result in the branchpoint being less than 43 nucleotides away from the intron’s 5’SS result in the skipping of the preceding exon or intron retention. **Figure S3.** Importance of features included in Introme. Feature importance is an estimate of the contribution of each variable to the model. Boxes are coloured and labelled according to the source of the feature annotated. **Figure S4.** Overview of the methods and data sources for each section. **Figure S5.** Performance of in silico splice-prediction tools on three validation data sets. Precision recall curves and the corresponding positions of the splice-altering variants in the dataset for A-B: ncVar, C-D: MFASS and E-F: Shiraishi. **Figure S6.** Performance of in silico splice-prediction tools on three validation data sets, broken down by regions.

**Additional file 3.** Review History.

## Data Availability

Introme is available at the Introme repository on GitHub (https://github.com/CCICB/introme) under the GNU General Public License v3.0 (GPLv3). The version of source code used in the manuscript is deposited in Zenodo [52]. All data generated or analysed during this study are included in this published article and its supplementary information files. Third party datasets used for the validation of Introme can be accessed through the following publications: MFASS [34], ncVar [49], and Shiraishi [50].
