# Supplementary material for: Introme accurately predicts the impact of coding and noncoding variants on gene splicing, with clinical applications
Source: Genome Biol. 2023 May 17;24:118. doi: 10.1186/s13059-023-02936-7 (PMC10190034; doi:10.1186/s13059-023-02936-7)
Supplement: Supplementary file 2 — Additional file 2: Figure S1. Comparison of in silico splice-prediction tools on validated non-splice-altering variants. Using an UpSet plot, variants identified here by one or more tools are in silico false positives. The CADD and CADD-splice tools contributed the largest number of false positives. Figure S2. Novel Functions incorporated into Introme. A: Position Weight Matricesfor the exonic splicing enhancer motifs from ESEfinder [15] showing relative representationof nucleotides at each position of the motif. B: PWM for the hnRNP A1 exonic splicing silencer motif [50]. C: Normal splicing pattern with the blocks representing the location of the exons. Creation of an AG motif greater or equal to 15 nucleotides away from the branchpoint results in the use of the new splice site. Creation of an AG less than 15 nucleotides away from the branchpoint results in exon skipping due to the AG exclusion zone. The consensus motifs for exon definition of the minor spliceosomeare different than the major spliceosome. Intronic deletions which result in the branchpoint being less than 43 nucleotides away from the intron’s 5’SS result in the skipping of the preceding exon or intron retention. Figure S3. Importance of features included in Introme. Feature importance is an estimate of the contribution of each variable to the model. Boxes are coloured and labelled according to the source of the feature annotated. Figure S4. Overview of the methods and data sources for each section. Figure S5. Performance of in silico splice-prediction tools on three validation data sets. Precision recall curves and the corresponding positions of the splice-altering variants in the dataset for A-B: ncVar, C-D: MFASS and E-F: Shiraishi. Figure S6. Performance of in silico splice-prediction tools on three validation data sets, broken down by regions. [file 13059_2023_2936_MOESM2_ESM.docx]

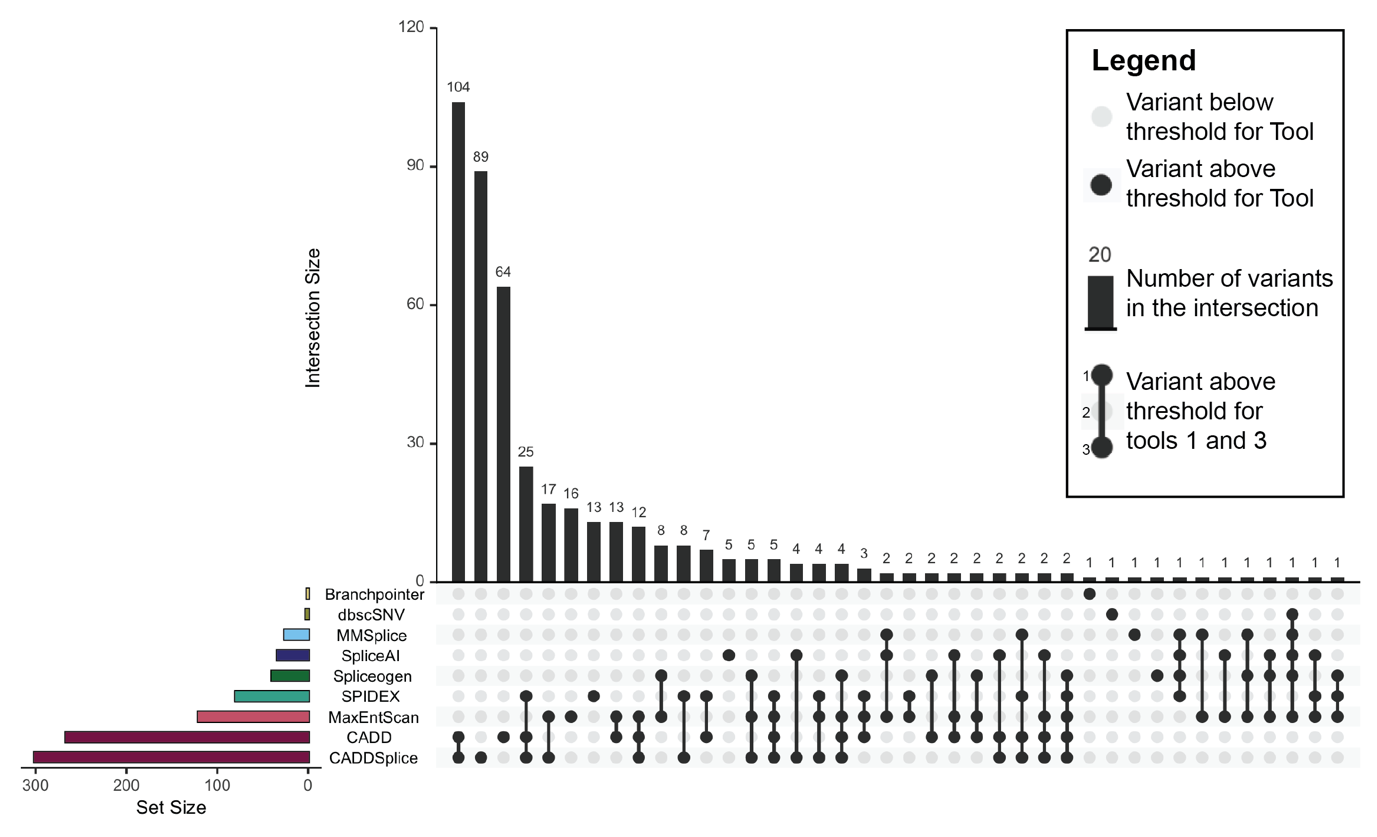


**Fig S1: Comparison of *in silico* splice-prediction tools on validated non-splice-altering variants.**

Using an UpSet plot (syntax is explained in Figure 2), variants identified here by one or more tools are *in silico* false positives. The CADD and CADD-splice tools contributed the largest number of false positives.


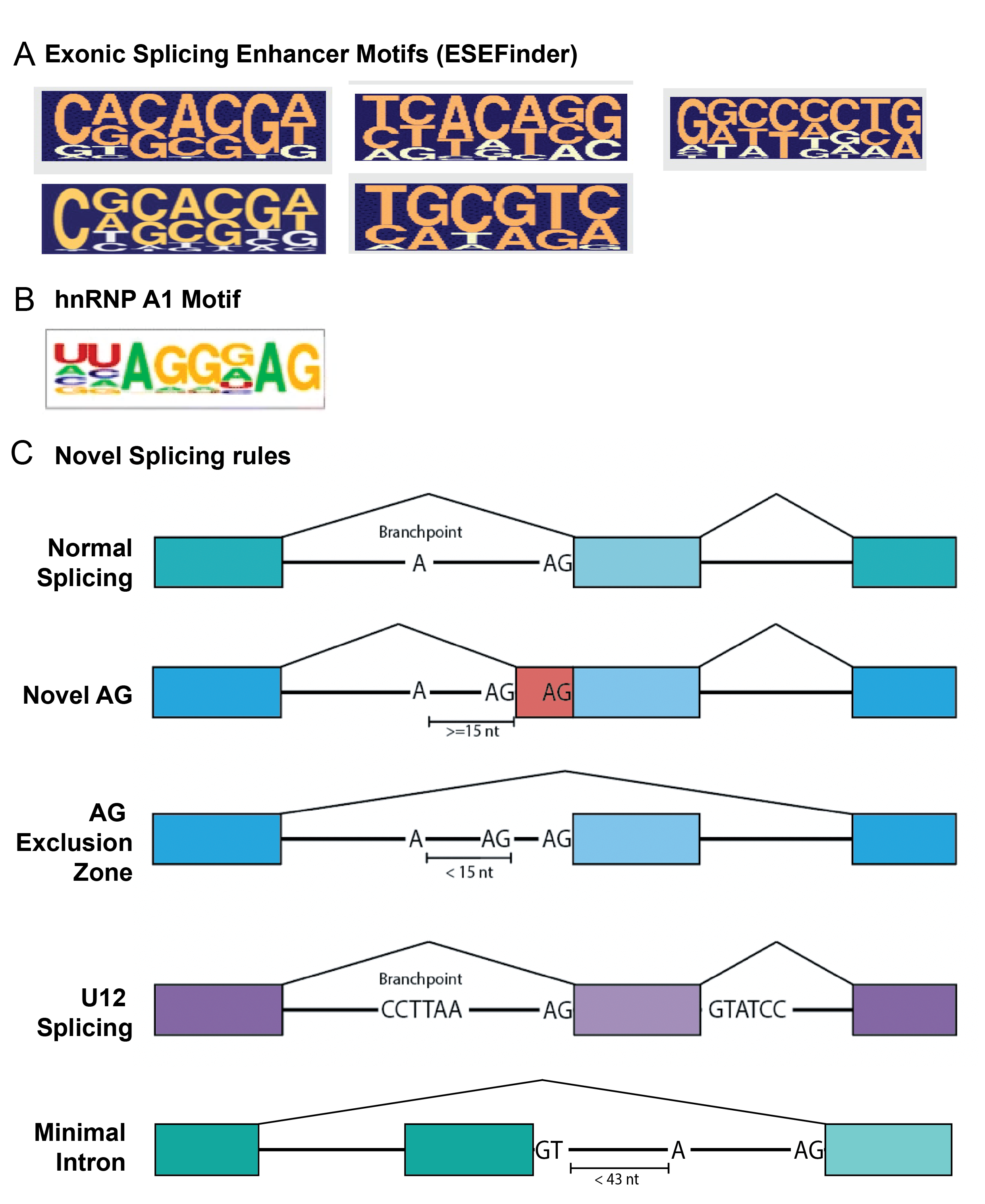


**Fig S2: Novel Functions incorporated into Introme.**

**A:** Position Weight Matrices (PWMs) for the exonic splicing enhancer motifs from ESEfinder [15] showing relative representation (height) of nucleotides at each position of the motif. **B:** PWM for the hnRNP A1 exonic splicing silencer motif [47]. **C:** Normal splicing pattern with the blocks representing the location of the exons. Creation of an AG motif greater or equal to 15 nucleotides away from the branchpoint results in the use of the new splice site. Creation of an AG less than 15 nucleotides away from the branchpoint results in exon skipping due to the AG exclusion zone. The consensus motifs for exon definition of the minor spliceosome (U12) are different than the major spliceosome (U2). Intronic deletions which result in the branchpoint being less than 43 nucleotides away from the intron’s 5’SS result in the skipping of the preceding exon or intron retention.


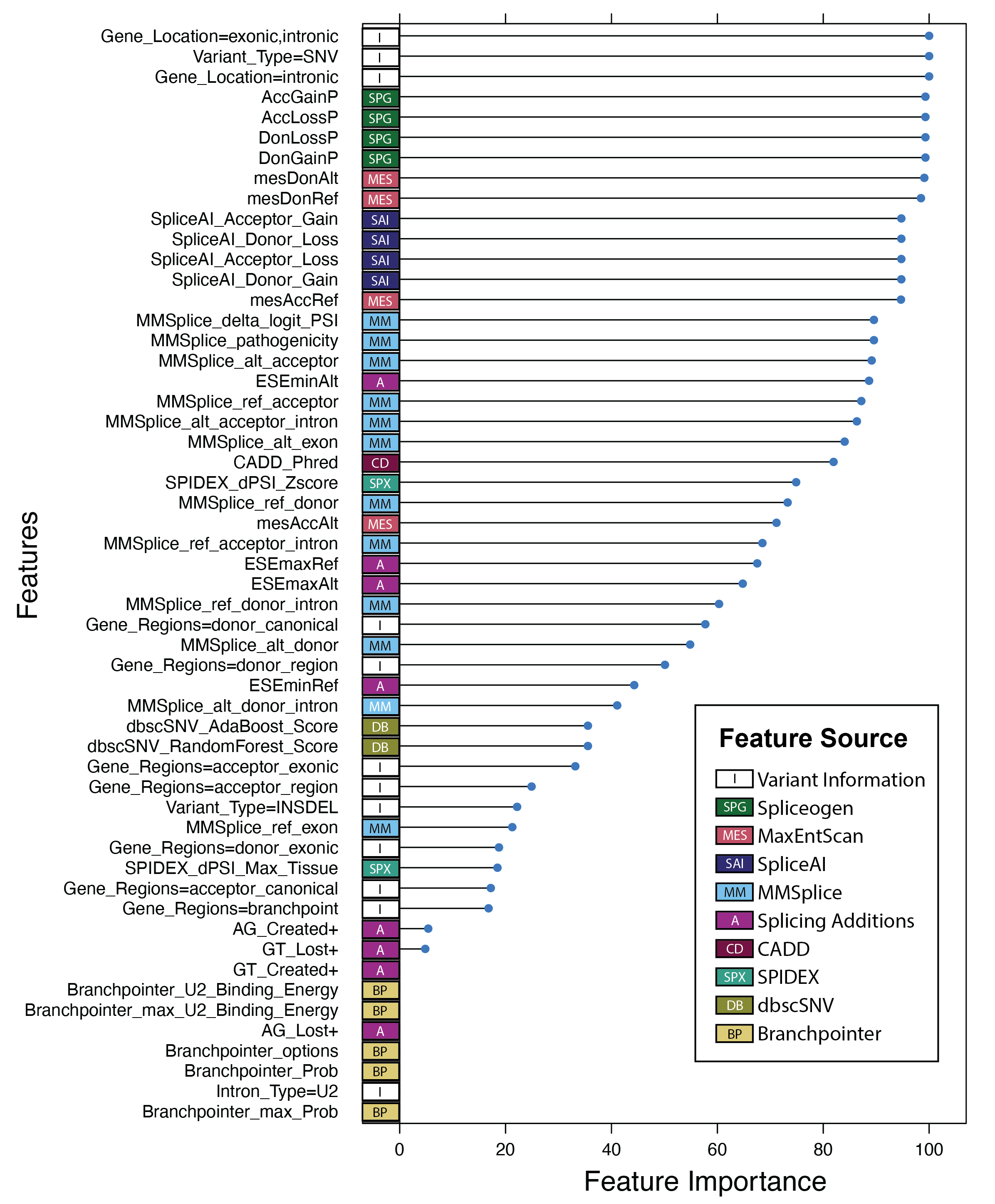


**Fig S3: Importance of features included in Introme.**

Feature importance is an estimate of the contribution of each variable to the model. Boxes are coloured and labelled according to the source of the feature annotated.


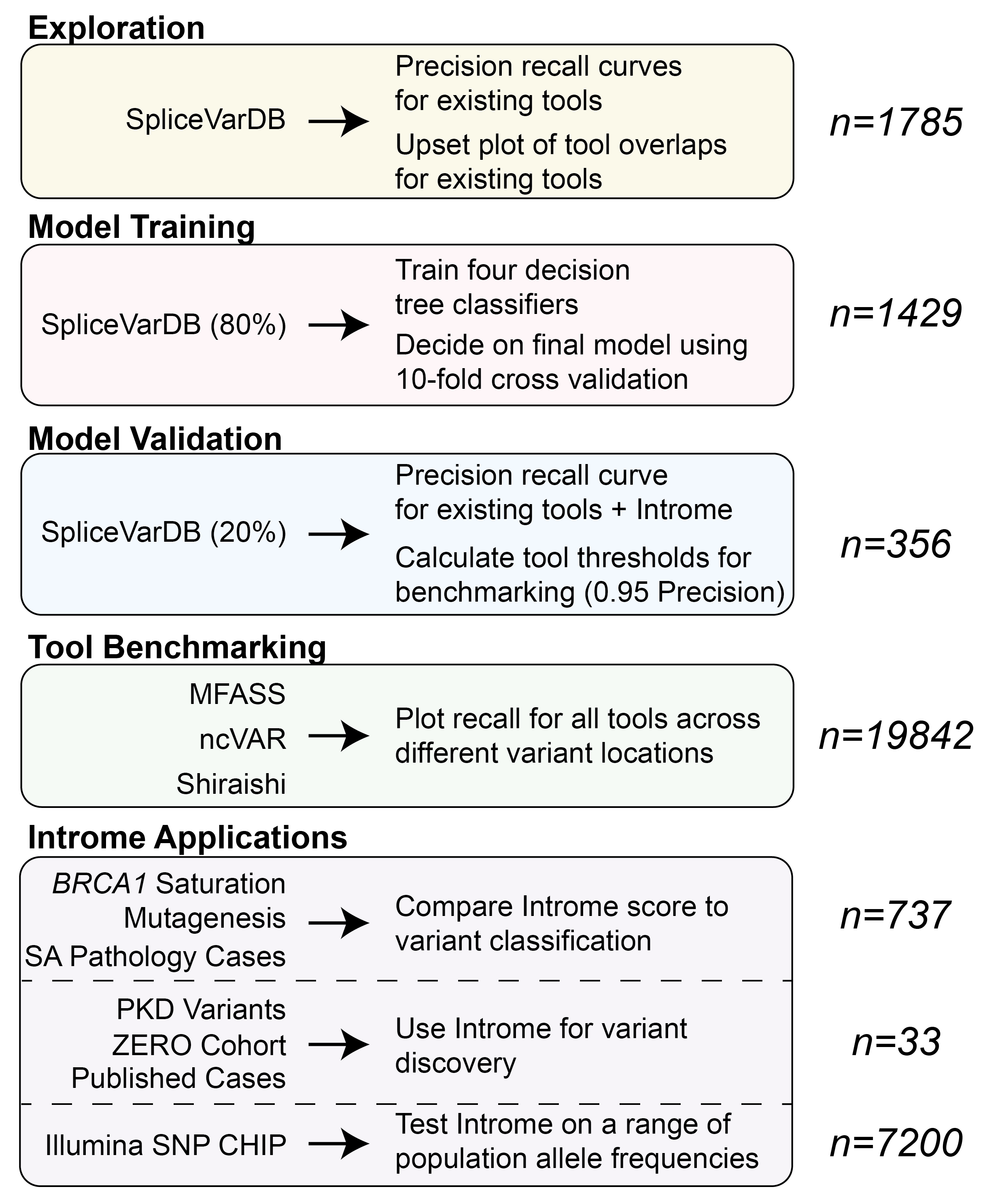


**Fig S4: Overview of the methods and data sources for each section.**


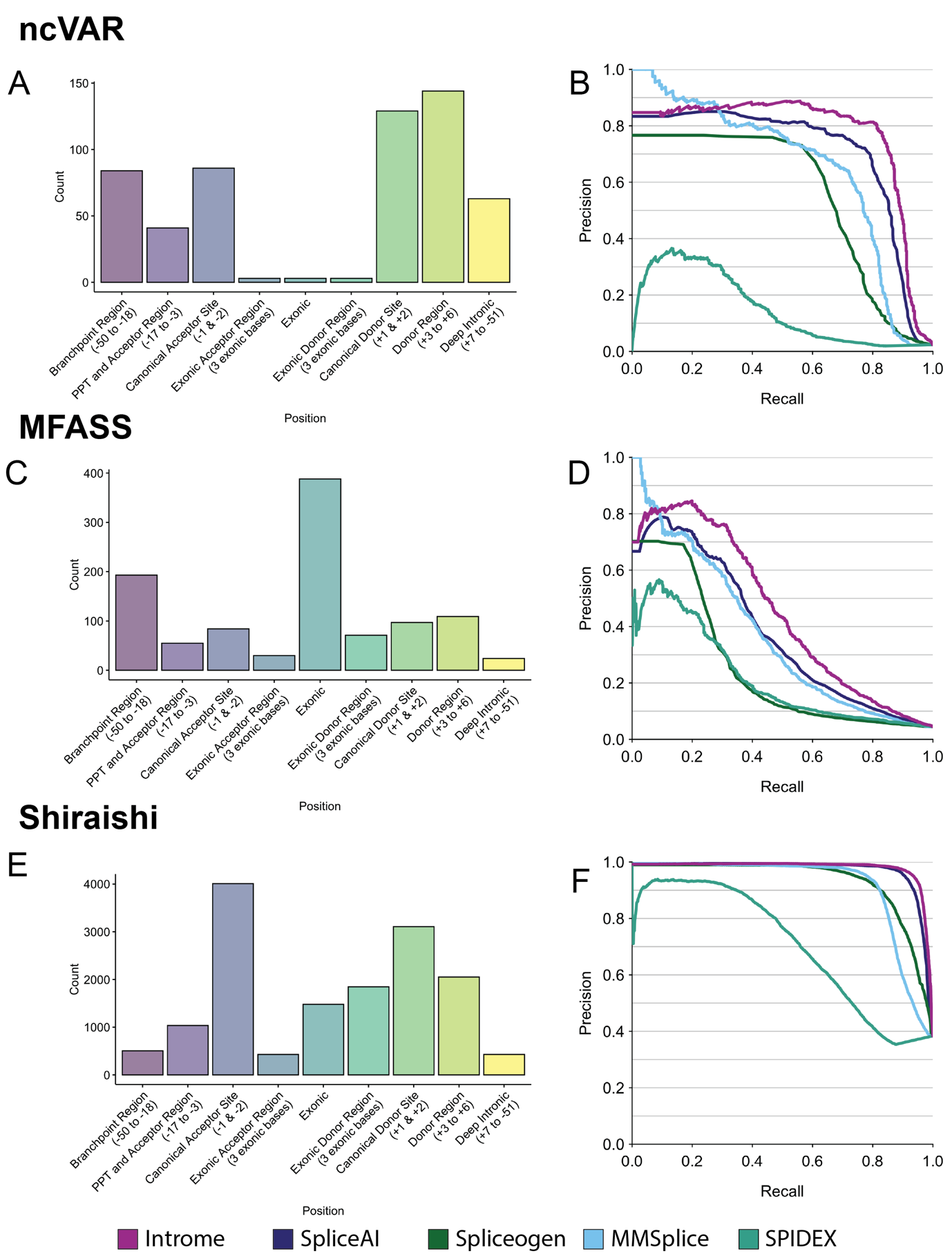


**Fig S5: Performance of *in silico* splice-prediction tools on three validation data sets (see Methods).**

Precision recall curves and the corresponding positions of the splice-altering variants in the dataset for A-B: ncVar, C-D: MFASS and E-F: Shiraishi.


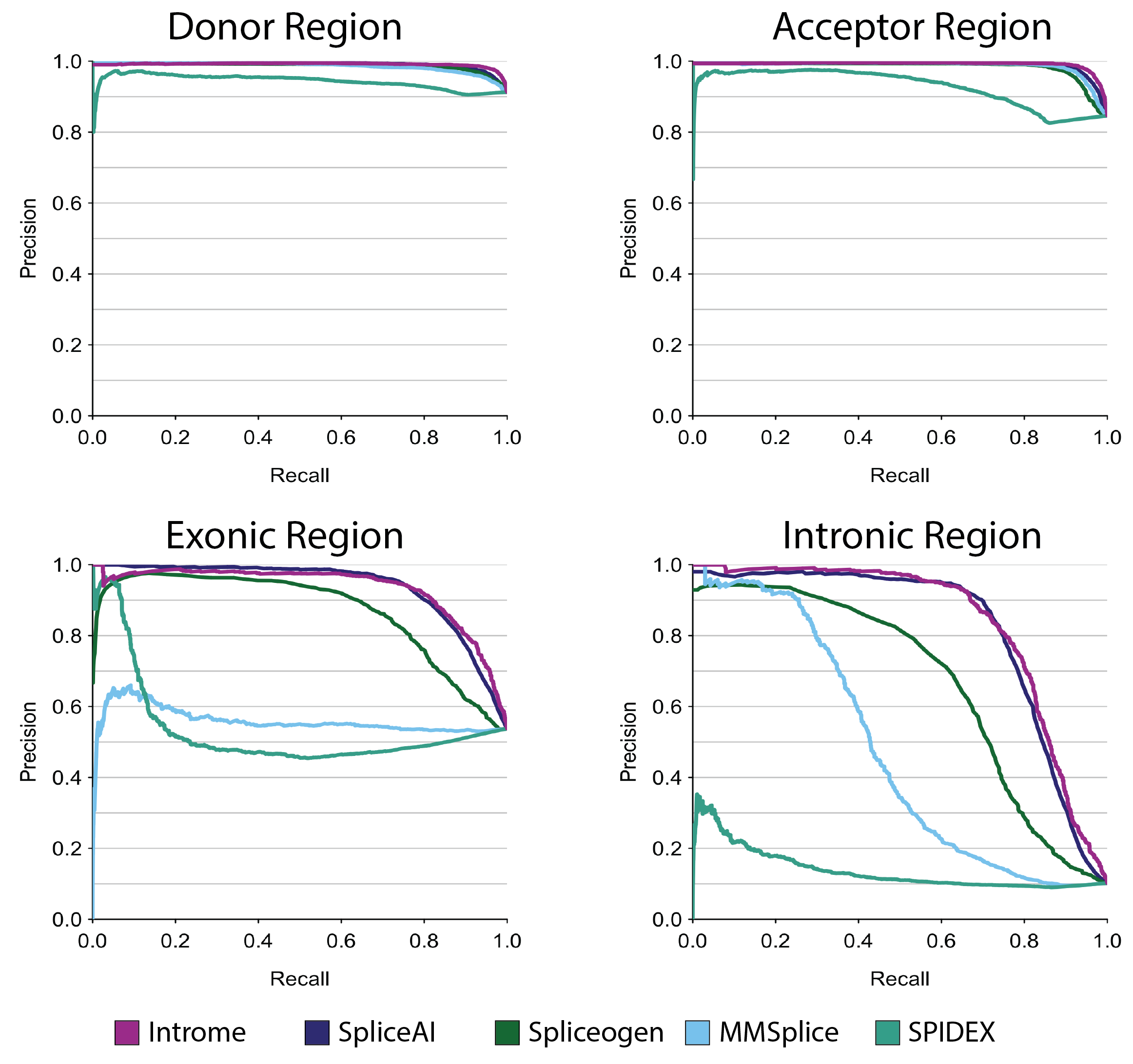


**Fig S6:** **Performance of *in silico* splice-prediction tools on three validation data sets, broken down by regions.**
