# Supplementary material for: Introme accurately predicts the impact of coding and noncoding variants on gene splicing, with clinical applications
Source: Genome Biol. 2023 May 17;24:118. doi: 10.1186/s13059-023-02936-7 (PMC10190034; doi:10.1186/s13059-023-02936-7)
Supplement: Supplementary file 3 — Additional file 3. Review History. [file 13059_2023_2936_MOESM3_ESM.docx]

Review History

**First round of review**

**Reviewer 1**

**Were you able to assess all statistics in the manuscript, including the appropriateness of statistical tests used?** No

**Were you able to directly test the methods?** No

**Comments to author:**

Sullivan et al. report a new computational tool, Introme, to predict the impact of human genetic variants on different classes of splicing events. Introme integrates other models with differing strengths and weaknesses, including SpliceAI, MMsplice and Spliceogen, with augmentation using a set of additional features in a decision tree model that overall performs better than the individual models on a set of experimentally validated variants represented by several different datasets. Several examples of various splicing event types are validated. The results are compelling and Introme appears to be the most effective tool described to date to predict the impact of variants on splicing in research and clinical diagnostic applications.

Main

(1) The authors are requested to provide a more detailed description of how Introme works in the main text. In the Methods section it should be clarified how the authors' model processes the outputs of SpliceAI, MMSplice and Spliceogen.

Minor

(1) The authors applied Introme only to rare variants, presumably because they are expected to have larger impact on splicing. Does Introme on average provide lower scores for common variants in comparison with rare disease-causing variants? Can the authors identify common variants with an unexpectedly large impact on splicing that potentially pre-dispose to disease?

(2) Introme predicts whether a variant is likely to affect splicing, but does not provide an estimation of the magnitude of the PSI change. The authors are requested to comment on this limitation and, as appropriate, discuss ways in which Introme could be improved in the future to address this limitation.

(3) From reviewing the authors' GitHub repository, it appears that Introme is only applicable to human genome sequence. It is recommended that the authors to make this clear in their manuscript. Also, it would be beneficial if the authors could ensure their method supports hg38. Currently, Introme only works with hg19, yet many researchers are now working with hg38.

(4) The authors highlight the interpretability of the decision tree model, but do not provide an interpretation. Which features/submodules are most critical for which types of variants?

(5) page 8. In regard to the last point, the novel features implemented should be described in more detail.

(6) Some definitions are vague: line 138: define 'impacted' more precisely

(7) line 217, 'essential splice regions'; Define 'donor' and 'acceptor' regions in the main text.

(8) Fig. 5A, explain ClinVar codes.

**Reviewer 2**

**Were you able to assess all statistics in the manuscript, including the appropriateness of statistical tests used?** Yes: I have assessed the statistics used

**Were you able to directly test the methods?** No

**Comments to author:**

The paper presents a software tool for prediction of splicing-altering variations from sequence. The paper is clearly argued and generally well conducted. The authors start by demonstrating empirically that different existing tools have complementary strengths and weaknesses, and then go on to derive an original tool which combines (using available machine learning approaches) different tool results as well as some additional information. The resulting tool appears to avoid the pitfalls of the state of the art, and seems to come as a usable software suite.

Overall, I think there is value in the work and the tool may be used by others. My reservations are technically minor:

- the level of novelty from the computational side is limited, this is an application of off-the-shelf ML to the output of existing methods. Whether this is a problem is an editorial decision but the level of interest in the methods development community will be low.

- I imagine given the translational focus we are here talking uniquely about human data (although actually I did not find such a statement). It might be interesting to see whether there is some transferability also to other organisms (mouse in particular) where pre-clinical studies might be done.

- the level of interest in the paper might be greater if the authors could present a more impressive case study where new biology can be learnt from using their tool (and which could not be learnt from other tools).

**Authors Response**

**Point-by-point responses to the reviewers’ comments:**

Reviewer reports:

*Reviewer #1: Sullivan et al. report a new computational tool, Introme, to predict the impact of human genetic variants on different classes of splicing events. Introme integrates other models with differing strengths and weaknesses, including SpliceAI, MMsplice and Spliceogen, with augmentation using a set of additional features in a decision tree model that overall performs better than the individual models on a set of experimentally validated variants represented by several different datasets. Several examples of various splicing event types are validated. The results are compelling and Introme appears to be the most effective tool described to date to predict the impact of variants on splicing in research and clinical diagnostic applications.*

We thank the reviewer for recognizing the strengths of Introme.

*Main Comments:*

(1) *The authors are requested to provide a more detailed description of how Introme works in the main text. In the Methods section it should be clarified how the authors' model processes the outputs of SpliceAI, MMSplice and Spliceogen.*

Thank you for this suggestion, we agree that adding more detail would strengthen the manuscript. Accordingly, we have updated details in both the “Introme scoring” methods section and Results section, as below. As discussed in response to minor comment #4, below, highlighting the feature importance also addresses this suggestion. The changes made are:

Results:

L174-210: “To develop a comprehensive splice-prediction tool, we first determined a comprehensive collection of features relevant for gene splicing. These included the scores from several leading splice prediction tools (as per Figure 1B), ESE/ESS motif strength, as well as sequence features such as whether the variant is in the branchpoint region, the AG Exclusion Zone (between the branchpoint and the 3’SS) [21], in a U12 intron (~0.5% of all introns are excised by the minor spliceosome instead of the major spliceosome) [22] and if the variant would lead to an intron smaller than the minimal length intron (distance between the branchpoint and the previous 5’SS is < 45nt) [23] (see Methods and Supplementary Figure 3). Following this feature selection, we selected a machine learning classification model that could handle missing data without imputation. We trained several models on the same training data, which were 80% (1429/1785) of the variants that we curated from the literature (n=940 with and n=489 without functional impact), and when assessing model performance using 10-fold cross validation, the C5.0 decision tree model [24] had the highest area under the receiver operating curve (auROC), and was thus selected as the basis for the Introme decision tree model (see Methods). The C5.0 learner has the added benefits of high-speed, and logical decision path (a series of nested if/else rules), which match the experience of investigators who may already be familiar with running each tool separately. The most important features in the model related to gene location, with scores from each of the different tools having varied contributions to the model (Supplementary Figure 4), reinforcing our intuition from Figure 1 that variant context is important for choosing the best splice-altering variant detection tool. Variants that affect splice branchpoint or U12 introns are rare, but biologically important, so despite these features having minimal overall importance, we retained these features in the model (Supplementary Figure 4). This model returns a score from 0–1 per variant reflecting the likelihood that the variant affects splicing (hereafter the Introme score). Different use cases require varying levels of sensitivity and specificity; therefore, and based on the model performance in the validation data we recommend two thresholds for Introme: the optimal balance between sensitivity and specificity (Youden’s Index) for Introme is at a threshold of 0.54, producing a sensitivity of 0.9 and a specificity of 0.95; when high specificity is required, a threshold of 0.75 results in a sensitivity of 0.8 and a specificity of 0.97.”

Methods: Introme Scoring:

L538-554: “Introme reports on a variant’s likelihood of altering splicing by combining predictions from splice-scoring tools and general variant information with machine learning methods (Figure 2). The input for Introme is a VCF file with GRCh37 or GRCh38 as the reference genome. Introme first subsets the file to contain only variants in the regions of interest (provided BED file or GTF). Remaining variants are then optionally filtered using user-provided quality metrics, annotated for population allele-frequency (both gnomAD [40] and MGRB [41]) using vcfanno [42], and by default, filtered on gnomAD_PopMax_AF ≤ 0.01. Variants are then annotated with CADD V1.3 (Combined Annotation Dependent Database) [43], SPIDEX V1.0 (Splicing Index) [6], dbscSNV V1.1 (Database of single nucleotide variants within splicing consensus regions) [44] and Branchpointer (gencode_v26) [45]. The filtered variants are scored using SpliceAI V1.3 with a distance setting of 1000 (-D 1000) and no masking (-M 0). MMSplice_MTSplice V2.2 and Spliceogen (v2.0) scores are obtained using default parameters. Introme’s novel functions (detailed below) are then applied. The scores obtained by each individual tool are reformatted to a VCF-like format that is compatible with vcfanno. All generated scores are then annotated onto the initial VCF, converted to a TSV file, and scored using the Introme model in R. All Introme features and inclusions to the final Introme score are listed in Supplementary Table 3.”

Additional small alterations were made, such as:

L130-135: “We identified, 1174 variants with and 611 variants without an effect on splicing across a range of rare genetic disease and cancer genes were used to train or test Introme (total n=1785; Supplementary Table 1).”

L167-168: “To assess the validity of this approach, we analysed the overlap of predictions on n=1174 known splice-altering variants (Figure 1B).”

L195-197: “We trained several models on the same training data, which were 80% of the variants that we curated from the literature (n=940 with and n=489 without functional impact), …”

L249-252: “The primary dataset used for this comparison was the remaining 20% of variants (n=356; n=234 with, and n=122 without functional impact) from the dataset that we curated from the literature and which were not included in the training set (see Methods).”

L501-503: “Models that could handle missing data predictions without imputation were selected and each model was trained on a dataset of 1,429 variants (training set), representing 80% of the 1,785 functionally validated splice-altering variants collected from the literature.”

Minor Comments:

(1) *The authors applied Introme only to rare variants, presumably because they are expected to have larger impact on splicing. Does Introme on average provide lower scores for common variants in comparison with rare disease-causing variants? Can the authors identify common variants with an unexpectedly large impact on splicing that potentially pre-dispose to disease?*

As the reviewer correctly points out, our objective with Introme was to find rare splice variants that are the cause of rare diseases or cancers; indeed, the default filter on Introme retains only rare variants with population maf ≤ 1%, though this can be altered by the user. We first made this change to the manuscript (L542-545):

“Remaining variants are then optionally filtered using user-provided quality metrics, annotated for population allele-frequency (both gnomAD [40] and MGRB [41]) using vcfanno [42], and by default, filtered on gnomAD_PopMax_AF ≤ 0.01”

To address the specific questions, we investigated how Introme performed on common polymorphisms represented on many genotyping arrays, stratified by different population allele frequencies, from rare to common. As expected, the rarer the polymorphism was, the more likely we were to see an impact on splicing. However, we did still see common polymorphisms with a predicted impact on splicing, including one with a strong association with chronic kidney disease from a GWAS. We have added this as a new case study, Figure element and Supplementary Figure, with the following text:

Methods in the section “Validation Methods” (L632-635):

“Variants used for allele frequency assessment were obtained from Illumina’s InfiniumCore-24v1.2 BeadChip and sorted into tranches based on gnomAD PopMax allele frequency [40]. A random set of 1200 variants for each tranche were selected to be scored using Introme (Supplementary Table 4).”

Results in the section “Assessing Introme’s utility for variant discovery” (L344-360):
“Finally, whilst the primary goal of Introme is to identify rare, splice-altering variants, we evaluated whether it could identify polymorphisms that might also affect splicing and potentially predisposition to common diseases. Thus, we ran Introme on a random selection of commonly genotyped polymorphisms chosen from six different population allele frequency tranches. As the polymorphisms became more common, they were less likely to be predicted by Introme as candidate splice-altering variants (Figure 5I). Of the 1200 randomly selected variants with a population allele frequency > 20%, nine were predicted to be splice-altering (Introme > 0.54). We manually inspected these for GWAS hits and found that one (rs11124542) was associated with Vitrin level in chronic kidney disease with hypertension and no diabetes (p-value 5x10^-12^, beta value 0.43 [0.31-0.55 CI]) [30]. While most polymorphisms are not expected to be causal variants, in this instance, the variant (*VIT*:c.679+11A>C) is in the gene that encodes *Vitrin*, and may be the causal variant through altering gene splicing, though further validation is required to validate this.”

Unexpectedly, many of the polymorphisms fall in genes not covered by MMSplice and SpliceAI, resulting in calls made only from Spliceogen, which has a high false positive rate when used alone. We improved Introme to mitigate this risk when used in this scenario, so we again thank the reviewer for this suggestion. We have addressed this limitation in the discussion (L399-402), as follows:

“When SpliceAI and MMSplice do not score a variant, which we saw primarily in the common variant analysis (Figure 5I), we observed a higher false positive rate. This was due to hard-coded region filters in these tools, so to address this, we changed the default behaviour of Introme to produce a score of zero in these cases.”

(2) *Introme predicts whether a variant is likely to affect splicing but does not provide an estimation of the magnitude of the PSI change. The authors are requested to comment on this limitation and, as appropriate, discuss ways in which Introme could be improved in the future to address this limitation.*

The reviewer correctly points out that Introme does not include PSI estimations. We re-reviewed our training data, and as we suspected, fewer than 10% of the functionally validated variants reported PSI. We currently lack the dataset to train Introme to report an accurate PSI prediction. We agree that this would be a useful future direction for Introme, and other splicing prediction tools, and anticipate that through more massively parallel splicing reporter assays, these datasets may appear over time. It is worth noting that MMSplice and SPIDEX do predict PSI, and these values are in the Introme output file; however, we do not assert their accuracy in this manuscript. We added the following text to the discussion to address this comment (L409-418):

“Further research to predict the functional impact (e.g., intron retention, exon skipping) of splice-altering variants and improved methods to identify splicing regulatory variants are required to further improve the utility of *in silico* splice predictors. In our training data, only 10% of variants had a reported quantitative value for the impact on splicing, namely the percent spliced in (PSI) value. The field needs to first gather more data linking genetic variants, to functional splice alterations and PSI, perhaps via massively parallel splicing assays [31]; this would then allow tools to be developed that better predict the precise functional impact of a splice-altering variant on the resulting mRNA transcript. In support of this goal, initiatives like SpliceVault are shedding light on rates of naturally occurring atypical splice site usage [32].”

(3) *From reviewing the authors' GitHub repository, it appears that Introme is only applicable to human genome sequence. It is recommended that the authors to make this clear in their manuscript. Also, it would be beneficial if the authors could ensure their method supports hg38. Currently, Introme only works with hg19, yet many researchers are now working with hg38.*

We have amended the manuscript to explicitly state that Introme can only be used for the analysis of human data (line 119). We have ensured that it is also explicitly stated on the GitHub readme.

Since the original submission of the article, we have released the hg38 compatible version of Introme on GitHub. We have updated the manuscript and the GitHub to reflect the applicability for hg38 (line 540).

(4) *The authors highlight the interpretability of the decision tree model, but do not provide an interpretation. Which features/submodules are most critical for which types of variants?*

The final method chosen for Introme, a C5.0 decision tree, can produce an interpretable decision tree diagram, highlighting the informative hyperparameters albeit one that is hard to represent in an A4 sized figure. To address this point, we have clarified the reference to the interpretability of the decision tree model in the following section:

L196-197: “The C5.0 learner has the added benefits of high-speed, and logical decision path (a series of nested if/else rules), which match the experience of investigators who may already be familiar with running each tool separately.”

We appreciate the interest of the reviewer in the feature importance of Introme and recognise the value reporting how Introme weights its features. We extracted the feature importance from the Introme decision tree and have represented them as a plot in Supplementary Figure 4. We have added the following section to the results:

L198-204: “The most important features in the model related to gene location, with scores from each of the different tools having varied contributions to the model (Supplementary Figure 4), reinforcing our intuition from Figure 1 that variant context is important for choosing the best splice-altering variant detection tool. Variants that affect splice branchpoint or U12 introns are rare, but biologically important, so despite these features having minimal overall importance, we retained these features in the model (Supplementary Figure 4).”

To assess the feature importance, we used VarImp from the caret package, which provides an estimate of overall variable importance for the entire model. As this assess performance of the whole model, it does not permit us to subset these findings to different variant types. It is intriguing that the most informative features capture variant regional effects, which we think addresses the intent of this question.

(5) *page 8. In regard to the last point, the novel features implemented should be described in more detail.*

We have added a new section in the methods to detail these new features. We also believe that the new additions relating to new Supplementary Figure 4 in response to comment #4 makes these novel features more understandable.

L556-L589: “*Novel Introme Functions:*

To further improve the ability of Introme to identify splice-altering variants, we developed several novel features focussed on known splicing rules (Supplementary Figure 3). To score variants affecting the five main exonic splicing enhancers, SRSF1, SRSF1(igM-BRCA2), SRSF2, SRSF5 and SRSF6, and the main exonic splicing silencer, hnRNP A1, we first obtained PWMs from ESEFinder [15] and experimental individual-nucleotide resolution crosslinking immunoprecipitation (iCLIP) data [46], respectively. Introme includes a PWM scoring script to assess the impact of a variant on each of these motifs.

To support the identification of splice-altering variants outside of the canonical splice regions, additional sequence features for the AG Exclusion Zone, U12 Spliceosome and Minimal Introns were implemented. Variants which create or remove an AG or GT sequence were flagged - this is particularly of use in the AG exclusion zone. Annotations from the Intron Annotation and Orthology Database [47] were added to support the identification of variants present in introns where splicing is mediated by the minor spliceosome (U12), which are often overlooked due to their rarity. When an intronic deletion occurs, the intron length is calculated to capture variants which result in introns under the minimal length (45 nucleotides) [23].

To enable SpliceAI scoring for insdels and multinucleotide variants, each complex variant was split into the corresponding deletion and insertion. The maximum score for each SpliceAI category was taken and used as the score for the complex variant. “

(6) *Some definitions are vague: line 138: define 'impacted' more precisely*

Mutations to an existing splice site can strengthen or weaken the site, so we changed ‘impacted’ to ‘altered’.

(7) *line 217, 'essential splice regions'; Define 'donor' and 'acceptor' regions in the main text.*

These are introduced on Line 73: “The main splicing motifs are the essential donor (5’) and acceptor (3’) splice sites at either end of the intron, the branchpoint and the polypyrimidine tract (PPT) [6].”

Line 220 renamed to “essential splice sites”

(8) *Fig. 5A, explain ClinVar codes.*

Figure 5A now has this sentence (L900-902) “Variants are coloured by their ClinVar pathogenicity classifications (P: Pathogenic; LP: Likely pathogenic; VUS: Variant of Uncertain Significance; B: Benign; LB: Likely Benign).”

Reviewer #2:

*The paper presents a software tool for prediction of splicing-altering variations from sequence. The paper is clearly argued and generally well conducted. The authors start by demonstrating empirically that different existing tools have complementary strengths and weaknesses, and then go on to derive an original tool which combines (using available machine learning approaches) different tool results as well as some additional information. The resulting tool appears to avoid the pitfalls of the state of the art, and seems to come as a usable software suite.*

*Overall, I think there is value in the work and the tool may be used by others. My reservations are technically minor:*

We thank the reviewer for the complements about the manuscript and Introme’s performance.

*Minor:*

(1) *The level of novelty from the computational side is limited, this is an application of off-the-shelf ML to the output of existing methods. Whether this is a problem is an editorial decision but the level of interest in the methods development community will be low.*

We accept this comment, as our goal with Introme was to develop a class-leading splice-altering variant prediction tool, leveraging our experience and novel training data, using well established machine learning techniques. We thank the reviewer for recognizing that the tool makes improvements in this regard over the state of the art, which we believe will be of value to the translational genomics research community, more than the pure computational methods development community.

(2) *I imagine given the translational focus we are here talking uniquely about human data (although actually I did not find such a statement). It might be interesting to see whether there is some transferability also to other organisms (mouse in particular) where pre-clinical studies might be done.*

We have amended the manuscript to explicitly state that Introme can only be used for the analysis of human data (Line 119). The tools that Introme leverages, namely dbscSNV, Branchpointer, Spidex, SpliceAI, MMSplice and Spliceogen are each developed specifically for humans. We agree that future work extending to model organisms would be useful but will require careful collection of new test and training data to account for the different complement of splicing factors and splice sites used in each organism and is thus beyond the scope of this manuscript.

(3) *The level of interest in the paper might be greater if the authors could present a more impressive case study where new biology can be learnt from using their tool (and which could not be learnt from other tools).*

We thank the reviewer for this suggestion. In the originally submitted manuscript, we glossed over the application of Introme to discover novel biology, so we have addressed this comment by adding three additional case studies, where Introme was used to find pathogenic variants that helped make diagnoses. These leverage our recent publications in *Nature Medicine*, *Am J Med Genet*, *Hum Mol Genet*. We have also added a new case study for polymorphisms that affect splicing and associated with disease. To this end, we have added a new elements to Figure 5 (C-E, I), and the following text:

L327-333: “During the development of Introme, we used it to discover splice-altering variants in several studies. We identified 28 reportable (pathogenic or likely pathogenic) mostly somatic splice-altering variants in a cohort of 247 patients with high-risk paediatric cancers [26], particularly in the well-established cancer genes *TP53*, *NF1* and *RB1* (Figure 5C-E). Additionally, we identified a 3’ UTR variant in *KLHL40* in a patient with nemaline myopathy 8 [27] and a missense variant in *YARS2* in a patient with a mitochondrial disorder known as MLASA2 [28], both of which Introme helped prioritise as candidate splice-altering variants. ”

The new text that accompanies Fig 5I is above in response to reviewer 1, minor comment 1.

**Second round of review**

**Reviewer 2**: I thank the authors for the comprehensive response, in particular for widening the case studies. I am now happy for the paper to go through.
